# Supplementary material for: Landscape of nuclear transport receptor cargo specificity
Source: Mol Syst Biol. 2017 Dec 18;13(12):962. doi: 10.15252/msb.20177608 (PMC5740495; doi:10.15252/msb.20177608)
Supplement: Supplementary file 1 — Appendix [file MSB-13-962-s001.pdf]

APPENDIX

Table of content

- Appendix Figure S1: Validation of experimental and statistical workflow
- Appendix Figure S2: Overlap and specificity within the nucleocytoplasmic transport system and validation of cargos in comparison to independent data
- Appendix Figure S3: Network propagated scores are biased in favor of hub nodes
- Appendix Figure S4: DNA-directed RNA Polymerase I/II/III

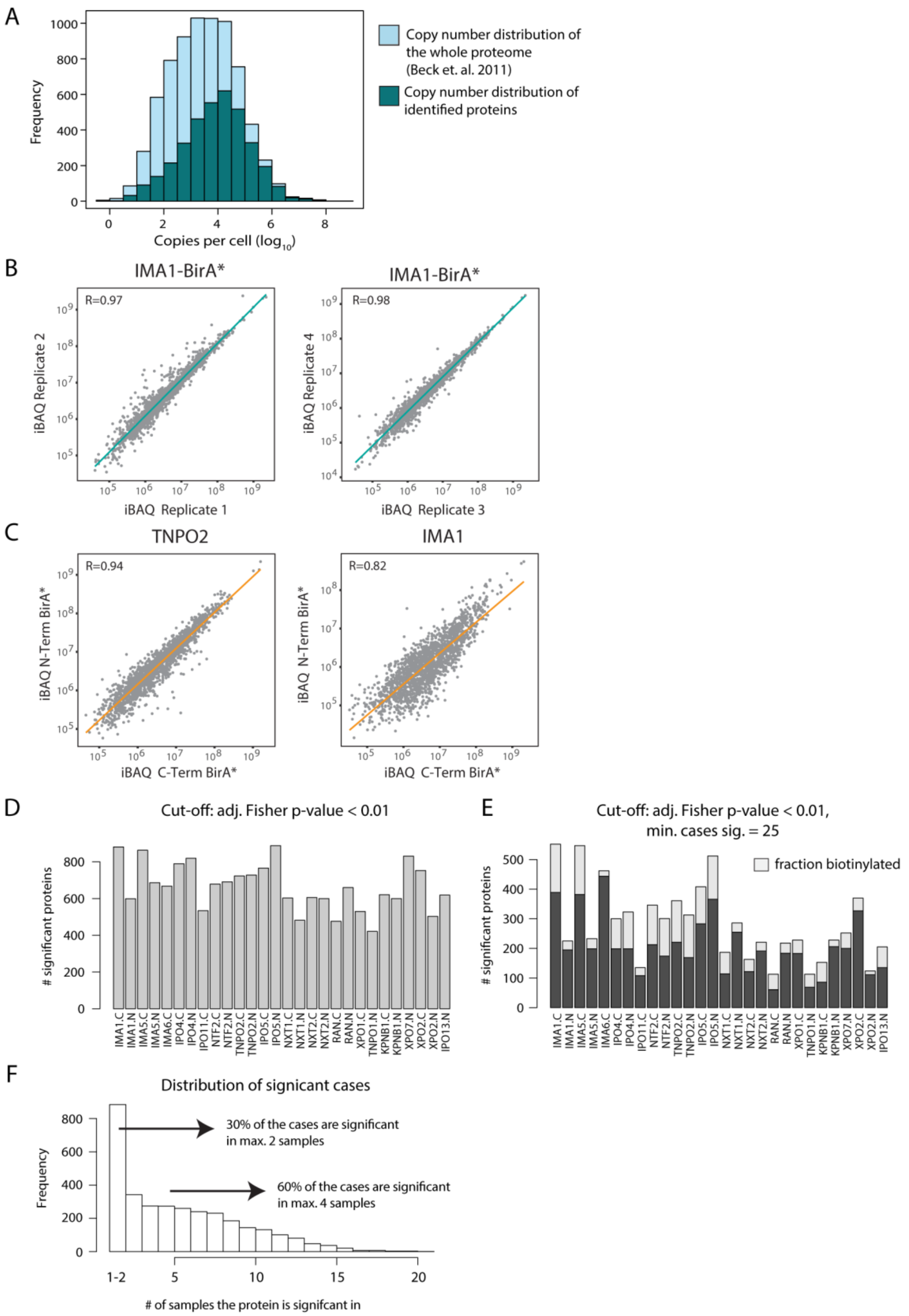

Appendix Figure S1: Validation of experimental and statistical workflow (A) Distribution of copy number of proteins identified in all experiments (excluding controls) based on a whole proteome study (Beck et al, 2011) in comparison to this work. Low abundant proteins are identified. (B) and (C) Correlation of protein abundances (iBAQ scores) of on-bead digested samples either between biological replicates (B) or between corresponding C- and N-terminally tagged NTRs (C). As expected, the correlation between biological replicates is higher. IMA1 and TPO2 are shown as examples. In case of IMA1 the correlation between N- and C-terminally tagged samples is reduced, likely because the IBB of N-terminally tagged IMA1 is sterically hindered. (D) Number of significant (adj. Fisher p-value < 0.01) proteins per experiment. (E) Number of significant proteins (as in D) identified to be significant enriched in at least 25 out of 27 comparisons with the number of identified biotinylated proteins. (F) Number of samples in which a protein has a significant specificity score. 30% of the significant cases occur in only 1 or 2 samples and 60% in max. 4 samples.

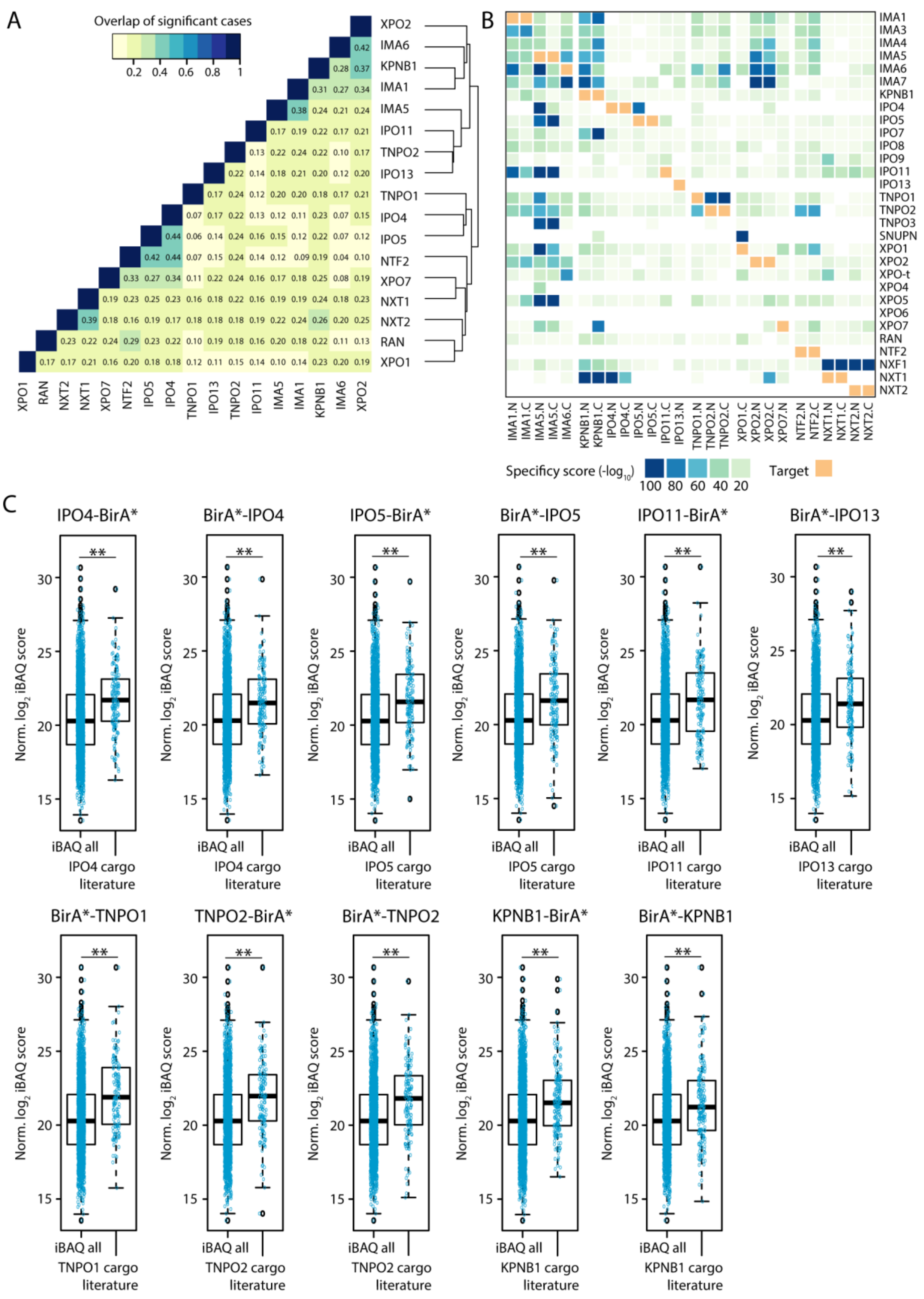

Appendix Figure S2: Overlap and specificity within the nucleocytoplasmic transport system and validation of cargos in comparison to independent data (A) Overlap of significantly enriched proteins across NTRs. Proteins significantly enriched (adj. Fisher p-value < 0.01) either in BirA*-NTR or NTR-BirA* experiments were combined when applicable. The heat map shows the fraction of significant cases shared between each pair of NTRs. (B) Heat map of reciprocal specificity scores across NTRs. A high degree of reciprocal interactions is observed among importin αs and between importin αs and XPO2. (C) Candidate cargos identified by Kimura et al. (Kimura et al, 2017) for IPO4, IPO5, IPO11, IPO13, TNPO1, TNPO2 and KPNB1 have significantly higher median abundances (iBAQ scores) in the respective samples analyzed by this study as compared to all identified proteins (** = Wilcoxon Signed-Rank Test P-value < 0.01).

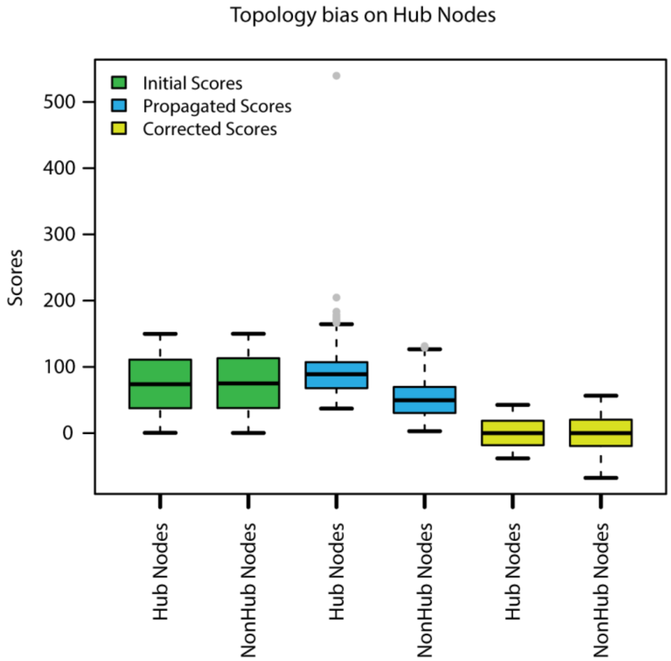

Appendix Figure S3: Network propagated scores are biased in favor of hub nodes From the human String protein-protein interaction network, the 10% nodes with highest degree were classified as 'hub nodes' and the 10% nodes with lowest degree were classified as 'non-hub nodes'. Random values between 0-150 equal to the number of nodes in the network were generated and applied on the network. Initially the hub and non-hub nodes have similar scores. These scores are propagated using α=0.5 and 31 iterations (till the scores converged, as performed for the NTR network). On average, the network propagated scores on hub nodes are higher compared to the propagated scores on non-hub nodes. Topology bias correction was therefore applied to balance network-propagated scores on hub and non-hub nodes.

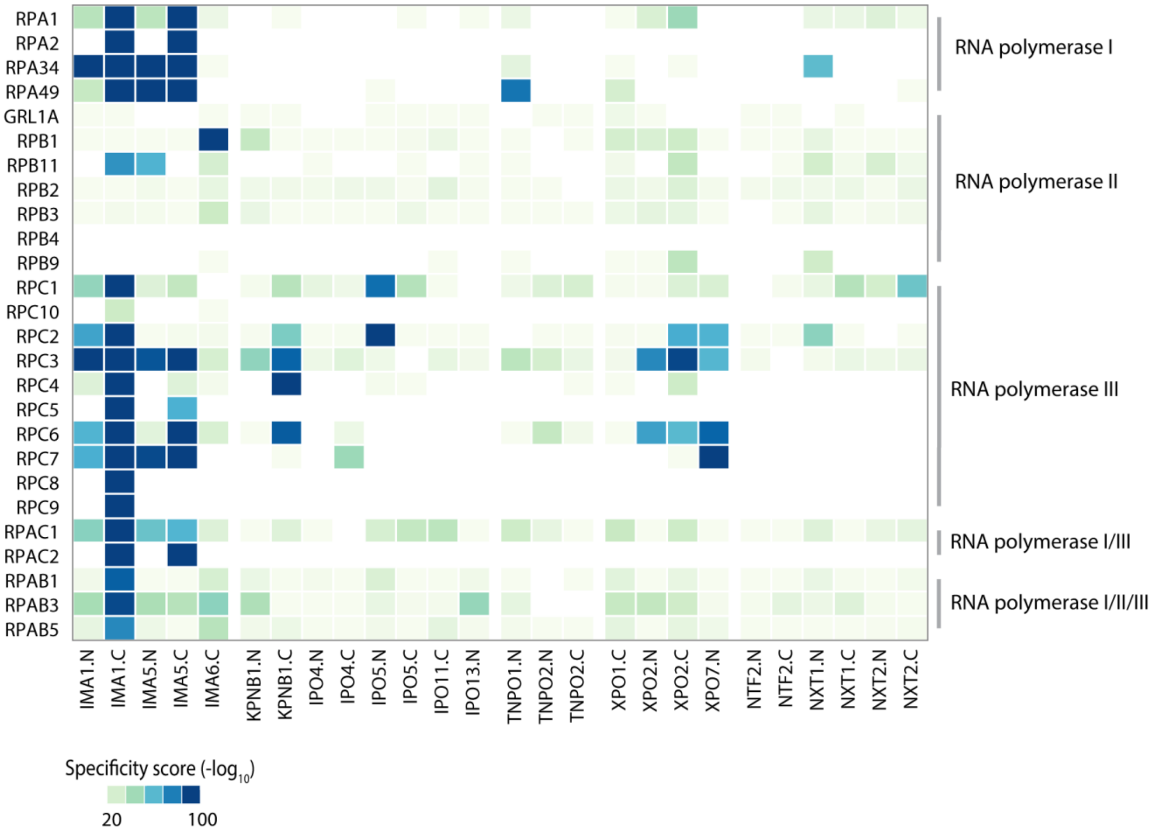

Appendix Figure S4: DNA-directed RNA Polymerase I/II/III Specificity scores of DNA-directed RNA polymerase I/II/III complex members are shown as a heat map across all experiments. The RNA polymerase I and III show a high specificity for IMA1-BirA* and partly IMA5-BirA*, while RNA polymerase II does not.
