# Supplementary material for: Landscape of nuclear transport receptor cargo specificity
Source: Mol Syst Biol. 2017 Dec 18;13(12):962. doi: 10.15252/msb.20177608 (PMC5740495; doi:10.15252/msb.20177608)
Supplement: Supplementary file 2 — Expanded View Figures PDF [file MSB-13-962-s002.pdf]

Expanded View Figures

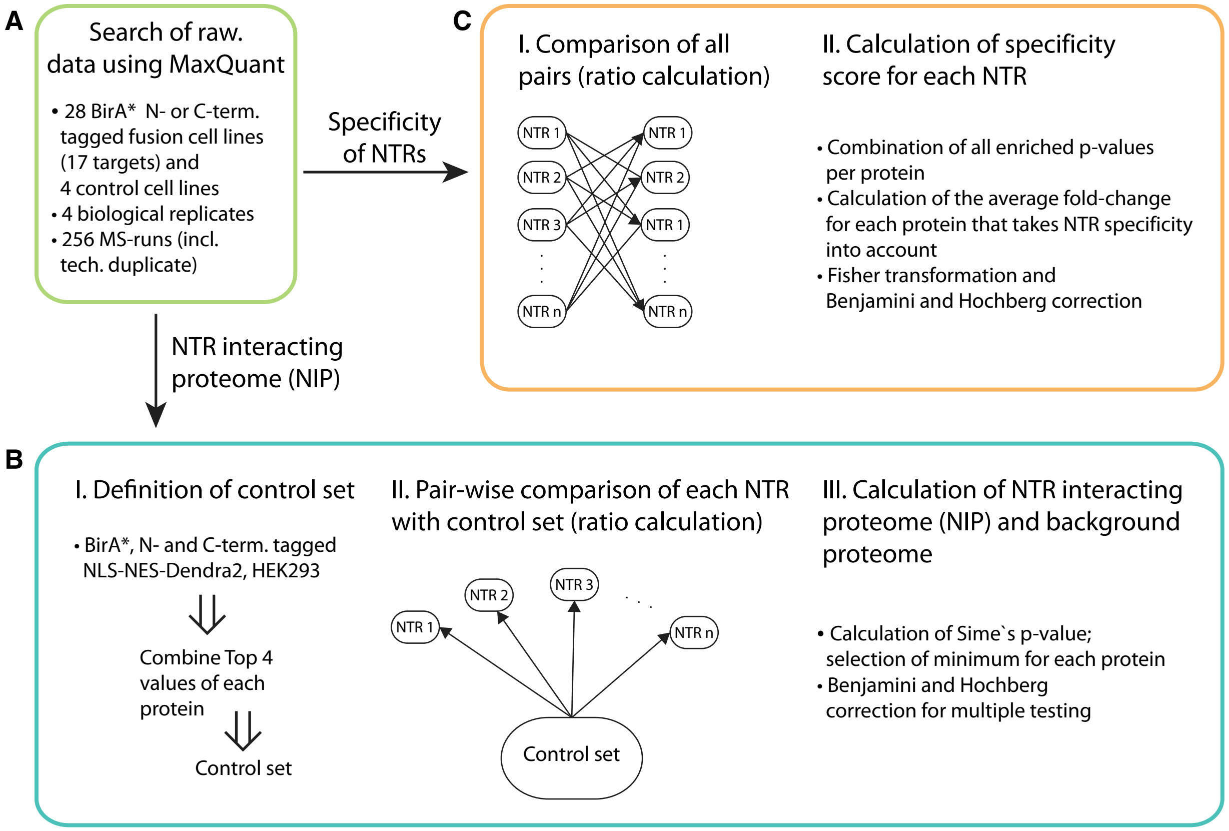

Figure EV1. Data analysis workflow for the NTR-interacting proteome (NIP) and NTR specificity.

- A Seventeen different transport factors and in total 28 N- and C-terminal BirA* fusion proteins and four control samples were measured in biological quadruplicate and technical duplicate. Protein intensities calculated using MaxQuant were used for further analysis.
- B The NTR-interacting proteome (NIP) and background proteome were calculated by quantification of NTR samples against the control set. The NIP does cover all NTR-interacting proteins regardless of how many NTRs they interact with.
- C Procedure to calculate protein specificity scores for each NTR. Proteins that interact with multiple NTRs are penalized (see Materials and Methods for details). The workflow for the direct identification of biotinylated peptides is not depicted in the figure.

Figure EV2. Effect of siRNA treatment and motif mutation on subcellular localization of cargos.

- A Automated detection of cells using the Hoechst staining as reference by CellProfiler. The overlaid numbers are the mean intensity in the cytoplasmic and nuclear area of anti-FLAG, which were used to exclude non-expressing cells.
- B iBAQ and specificity scores obtained with BirA*-EIF3D as scatter plot; members of eukaryotic translation initiation factor 3 and NTRs are highlighted. Importin βs are significantly enriched for EIF3D.
- C Specificity score for EIF3D obtained with various NTR-BirA* fusion proteins. IPO7 and IPO8 identified in the reciprocal BirA* AP were not part of the initial data set.
- D Subcellular distribution of the BirA*-EIF3D upon siRNA treatment against IPO4, IPO5, IPO11, TNPO2, and a negative control (scrambled siRNA). IPO5 and IPO11 induce the strongest shift of EIF3D toward the cytoplasm.
- E Quantification of the ratio of nucleoplasmic to cytoplasmic (N/C) distribution of EIF3D upon siRNA treatment (**Wilcoxon signed-rank test *P*-value < 0.01).
- F Subcellular distribution of BirA*-EIF3D upon removal and replacement of a DE-rich motif predicted by DILIMOT with a linker. The deletion but not substitution of the predicted DE-rich motif leads to a nucleoplasmic retention.
- G Quantification of the N/C ratio upon DE-rich motif removal (**Wilcoxon signed-rank test *P*-value < 0.01).
- H Bar chart of knock-down efficiency after siRNA mediated depletion of selected NTRs (IMA1, IMA5, IPO11, IPO4, IPO5, and TNPO2). Transcript levels were quantified by qPCR, and they are expressed as knock-down efficiency. The knock-down efficiency ranges from 39% (IMA5) to 94% (IMA1) after 3 days of treatment. Error bars indicate standard deviation. *N* = 3.

Data information: Scale bar, 10 μm. Boxplots: the upper and lower limit of the box indicate the first and third quartile, respectively, and whiskers extend 1.5 times the interquartile range from the limits of the box.

© 2017 European Molecular Biology Laboratory

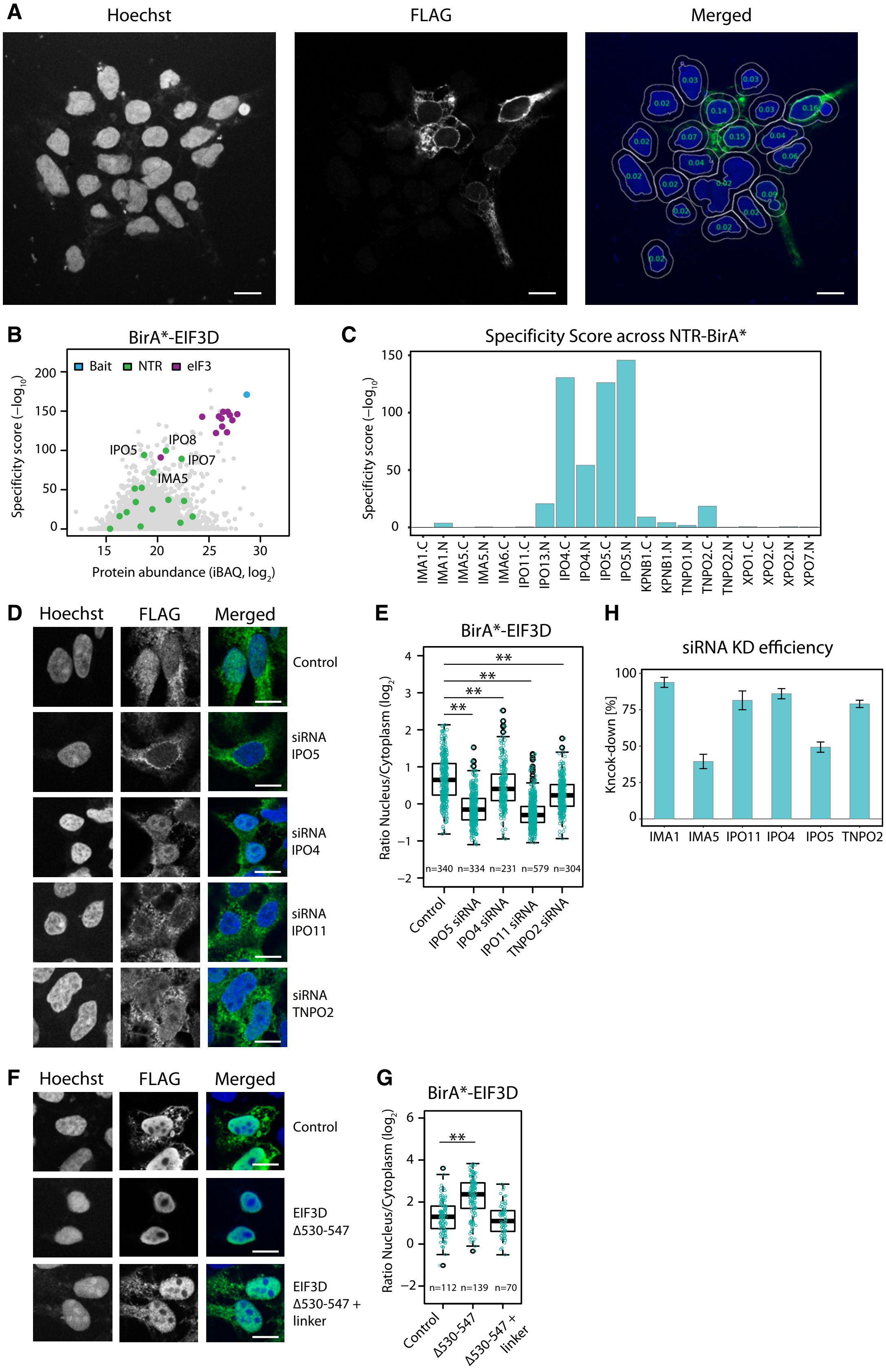

Figure EV2.

© 2017 European Molecular Biology Laboratory

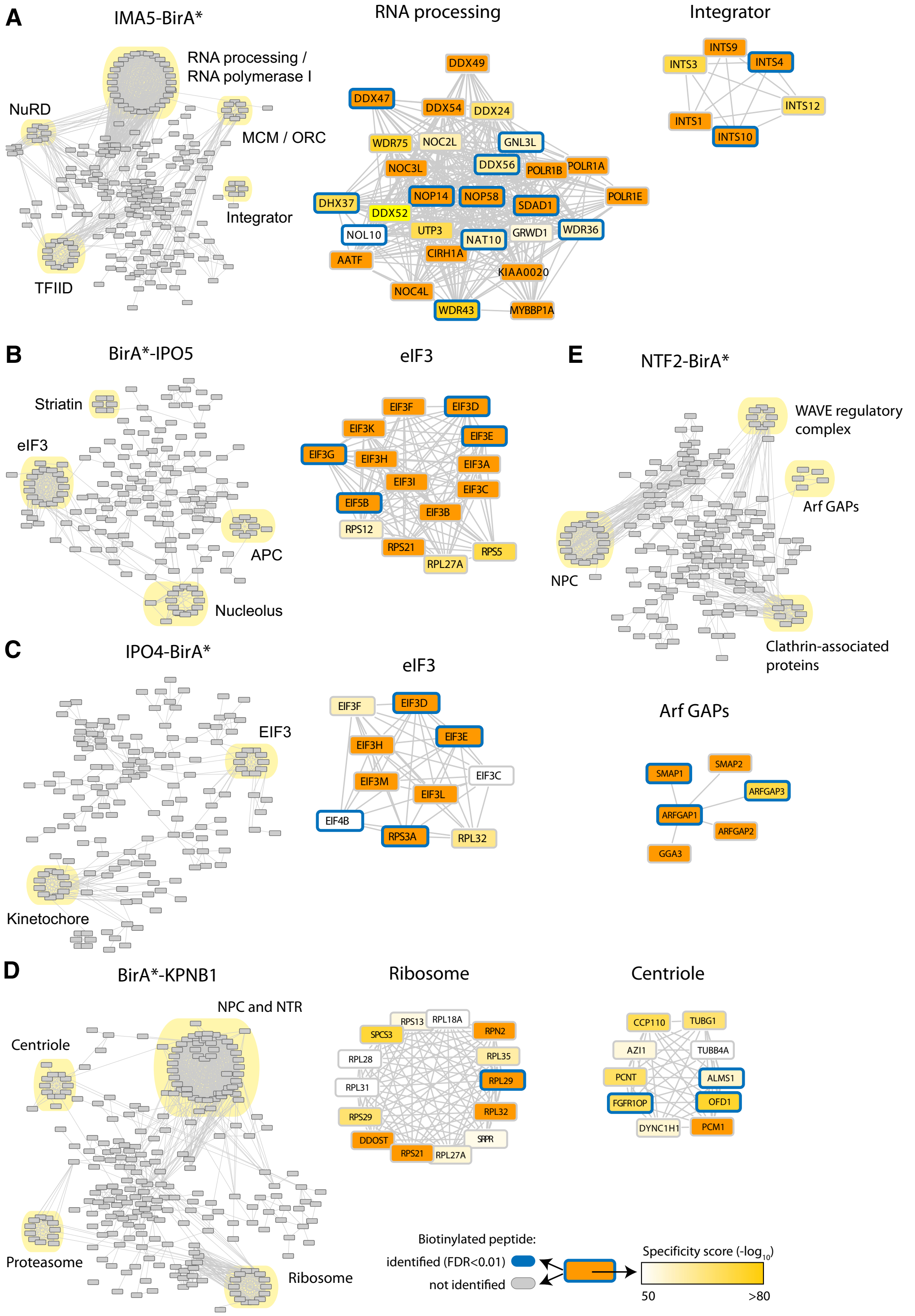

Figure EV3. Network analysis.

A–E Network analysis of the top 2% enriched proteins for IMA5-BirA* (A), BirA*-IPO5 (B), IPO4-BirA* (C), BirA*-KPNB1 (D), and NTF2-BirA* (E). Selected subnetworks are shown with the specificity score (color gradient) and detected biotinylated peptides (blue frame). MCM (minichromosome maintenance), ORC (origin recognition complex), eIF3 (eukaryotic translation initiation factor 3), APC (anaphase-promoting complex), Arf GAP (ADP-ribosylation factor GTPase-activating proteins), WAVE (WASP-family verprolin homologous protein).

© 2017 European Molecular Biology Laboratory

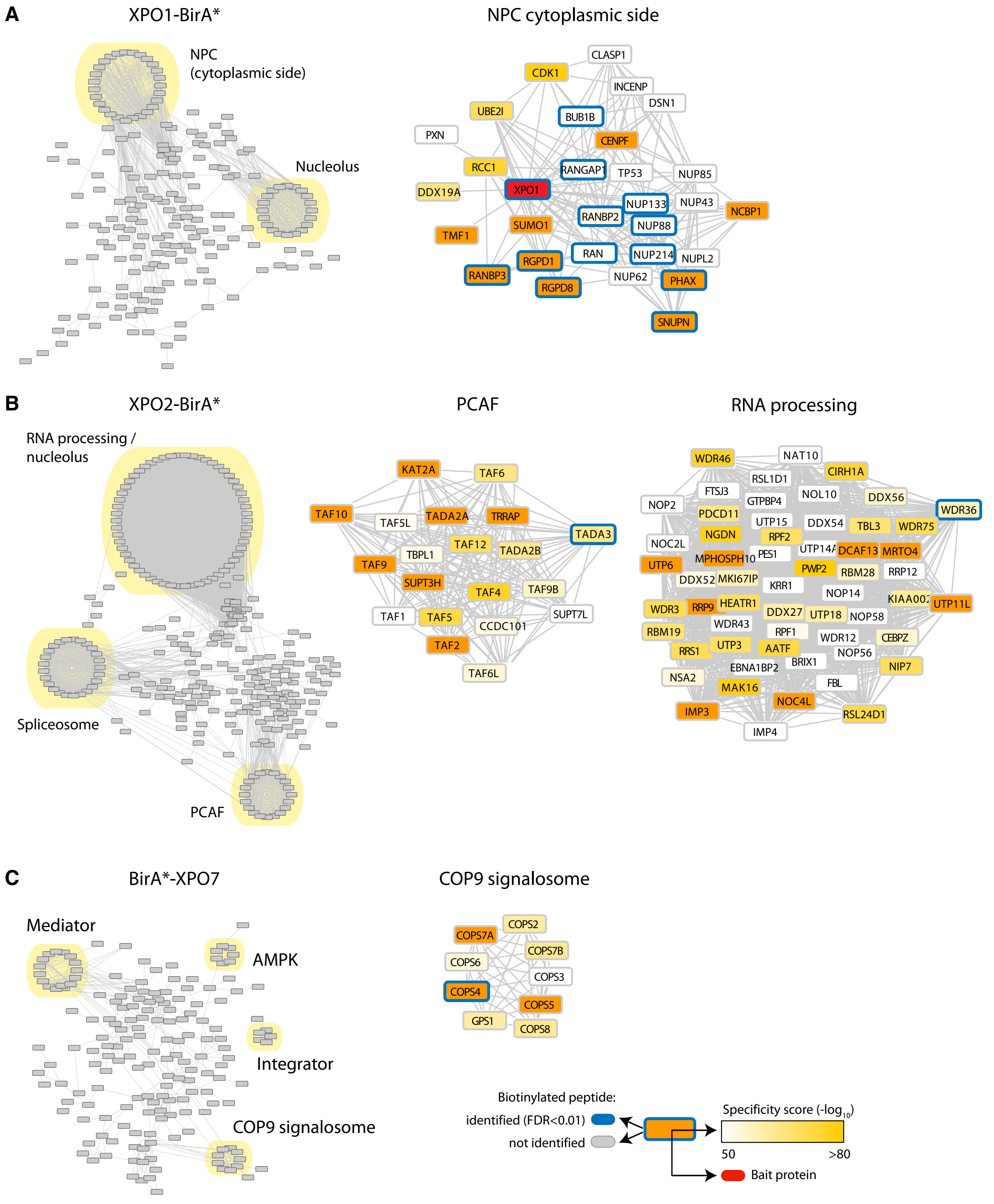

Figure EV4. Network analysis.

A–C Same as Fig EV3 but for XPO1-BirA* (A), XPO2-BirA (B) and BirA*-XPO7 (C). PCAF (p300/CBP-associated factor), AMPK (AMP-activated protein kinase), COP9 (constitutive photomorphogenesis 9).

© 2017 European Molecular Biology Laboratory

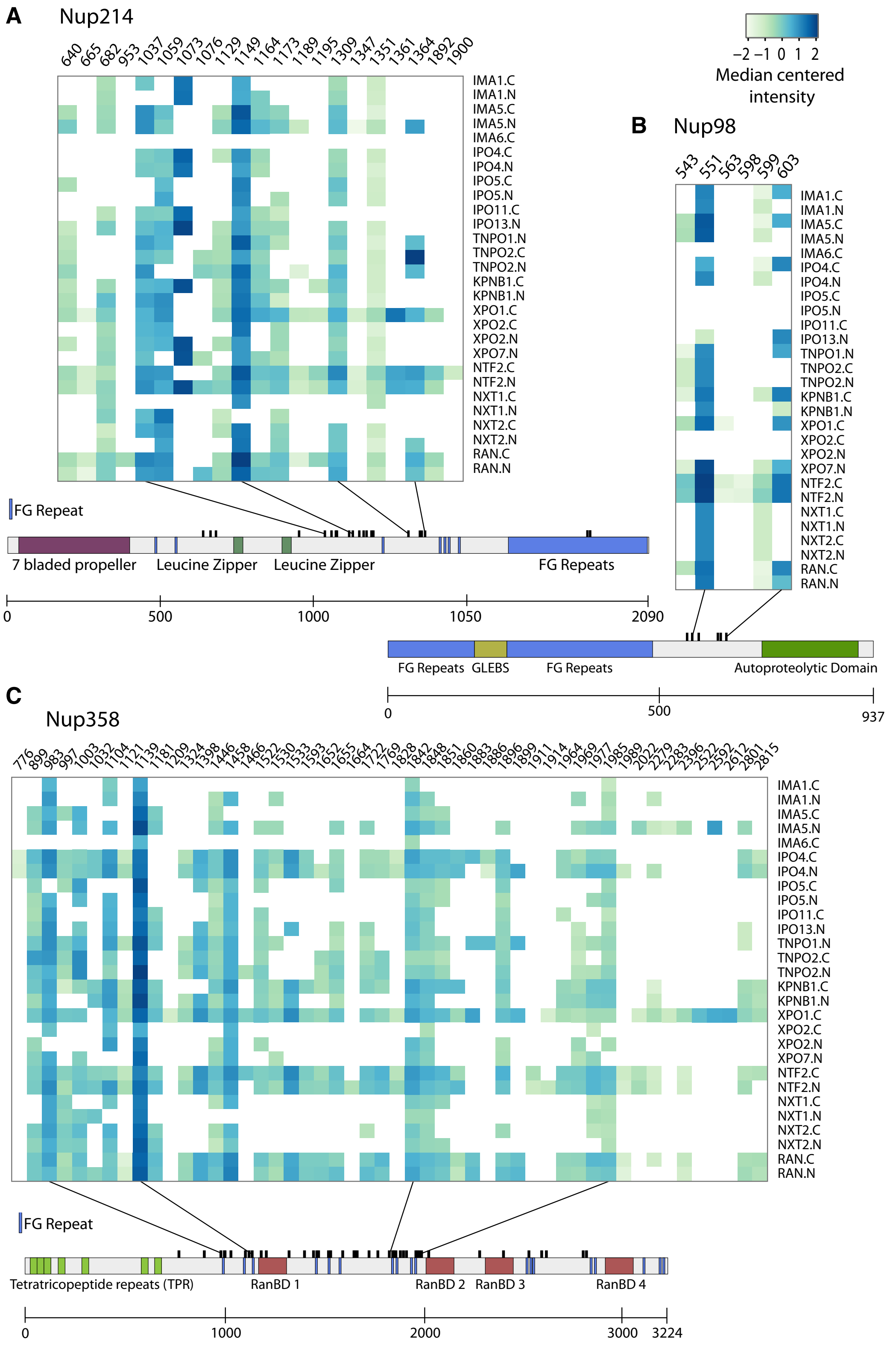

Figure EV5. Direct identification of biotinylated peptides suggests that NTRs preferentially bind specific FG-Nups *in situ*.

A–C Same as Fig 8 but for Nup214 (A), Nup98 (B), and Nup358 (C). Structural domains like seven bladed propeller, leucine zippers, autoproteolytic domain, tetratricopeptide repeats (TPR), and Ran-binding domains (RanBD) are highlighted as well as FG-repeats.

© 2017 European Molecular Biology Laboratory
